# Development of *Dirofilaria immitis* within the mosquito *Aedes* (*Finlaya*) *koreicus*, a new invasive species for Europe

**DOI:** 10.1186/s13071-015-0800-y

**Published:** 2015-03-23

**Authors:** Fabrizio Montarsi, Silvia Ciocchetta, Gregor Devine, Silvia Ravagnan, Franco Mutinelli, Antonio Frangipane di Regalbono, Domenico Otranto, Gioia Capelli

**Affiliations:** Istituto Zooprofilattico Sperimentale delle Venezie, Viale dell’Università, 10; 35020 Legnaro, Padua, Italy; Mosquito Control Laboratory, QIMR Berghofer Medical Research Institute, Brisbane, Queensland Australia; Queensland University of Technology, Brisbane, Queensland Australia; Dipartimento di Medicina Animale, Produzioni e Salute, Università degli Studi di Padova, Viale dell’Università, 16; 35020 Legnaro, Padua, Italy; Dipartimento di Medicina Veterinaria, Università degli Studi di Bari, Valenzano, 70010 Bari, Italy

**Keywords:** *Aedes koreicus*, Invasive mosquitoes, *Dirofilaria immitis*, Vector competence, North-eastern Italy

## Abstract

**Background:**

Over the recent decades, container-breeding mosquito species belonging to the genus *Aedes* have frequently been recorded far from their place of origin. *Aedes koreicus* was first reported in north-eastern Italy in 2011, in a region endemic for *Dirofilaria immitis,* the agent of canine heartworm disease. The vector competence of *Ae. koreicus* for *D. immitis* was here tested under laboratory conditions, by infecting mosquitoes with a local strain of *D. immitis*.

**Methods:**

Blood containing 3000 microfilariae/ml was offered to 54 mosquitoes (T group) while 29 were left as a control (C group). Mosquitoes killed at scheduled days post infection (dpi) and naturally dead were divided in head, thorax and abdomen and examined for *D. immitis* larval stages by dissection under a microscope and molecularly.

**Results:**

Of the 45 engorged mosquitoes in T, 32 (71.1%) scored positive for *D. immitis* larval stages. L3 were found as early as 8 dpi in the Malpighian tubules and then in the thorax, salivary glands, palp and proboscis. At the end of the study a total of 18 mosquitoes developed L3 giving an estimated infection rate at 12 dpi of 68.2% and a vector efficiency index of 25.2%. The rate of mortality in T group within the first 9 days post infection was significantly higher in T group (47.6%) than in C group (8.3%) (p < 0.01). The concordance between microscopy and PCR was high (0.8-0.9), however, a positivity for *D. immitis* in the head was found molecularly at 13 dpi, three days before microscopy.

**Conclusions:**

*Aedes koreicus*, a new invasive species for Europe, is most likely a competent vector of *D. immitis* being of potential relevance in the natural cycle of the parasite. This poses a new threat for animal and human health in endemic areas for dirofilariosis and enhances the risk of spreading the infection in previously non-endemic areas. These results stress the importance of active surveillance and control strategies to minimize the risk of introduction of invasive alien species.

**Electronic supplementary material:**

The online version of this article (doi:10.1186/s13071-015-0800-y) contains supplementary material, which is available to authorized users.

## Background

Over the recent decades, container-breeding mosquito species belonging to the genus *Aedes* (Meigen) have frequently been recorded far from their place of origin [1]. Some invasive species of *Aedes* are capable of transmitting viral, bacterial or parasite pathogens of public health concern when they establish in previously non- endemic geographic areas [2,3]. A key example is the tiger mosquito *Aedes albopictus* (Skuse) (syn. *Stegomyia albopicta*) that was first imported to Italy in the early 1990s [4], being later responsible for an outbreak of chikungunya virus in 2007 [5].

The latest invasive container-breeding mosquito introduced in Europe was *Aedes* (*Finlaya*) *koreicus* (Edwards), a species native of Korea, China, Japan [6,7] and of the Asian part of Russia [8]. Since its first report in Belgium in 2008 [9], this mosquito has been identified in north-eastern Italy in 2011 [10] and in the south of European Russia in 2013 [11]. While in Belgium the mosquito is localized in a very restricted area of about 6 km^2^ [12], in Italy it has colonized more than 3000 km^2^ with an expanding trend [13].

Scientific information on the biology, ecology and vector competence of *Ae. koreicus* is lacking, and compounded by the fact that this mosquito species has long been misidentified as *Aedes japonicus japonicus* (Theobald), a species morphologically similar which lives in sympatry with the former [14].

The dog heartworm *Dirofilaria immitis* (Filarioidea, Onchocercidae), a filarial nematode primarily affecting dogs and less frequently cats and humans, is endemic in northern Italy, particularly along the Po River valley and the surrounding flat areas [15,16]. The prevalence of *D. immitis* in stray dogs living in the same geographical area where *Ae. koreicus* most likely established for the first time*,* has been reported as high as 67% [17]. Accordingly, recent surveys on mosquito species potential vectors of this filarioid (i.e., *Culex pipiens* L., *Aedes (syn. Ochlerotatus) caspius* (Pallas), *Aedes vexans* (Meigen)) demonstrated that infected mosquitoes are widespread all over the region [18] and that dogs and humans are most likely exposed to infected mosquitoes as often as every four nights and 14 nights, respectively [19]. Additionally, *Ae. albopictus* has been confirmed as a natural vector of dirofilariosis in Italy [20], whereas *Ae. koreicus* was found as a vector of *D. immitis* in a single laboratory trial carried out in Korea [21]. Nonetheless, in the latter study, a clear discrimination between *Ae. koreicus* and *Ae. japonicus japonicus* was not possible, since the two species were only clearly described in the 1950s [14].

Therefore, the vector competence of *Ae. koreicus* for *D. immitis* was here assessed under laboratory conditions, by infecting a mosquito colony derived from specimens collected from new geographic foci in north-eastern Italy, with a local strain of *D. immitis* from an autochthonously infected dog.

## Methods

### *Aedes koreicus* colony

The colony of *Ae. koreicus* originated from larvae collected from a field site (Province of Belluno, north-eastern Italy) where the presence of *Ae. albopictus* was not previously reported [13]. Larvae were kept in the same water as they were collected from until adult emergence. A subset (about 10% of the larvae and adults) were confirmed as *Ae. koreicus* by morphology and PCR as described elsewhere [10,22]. No other species were present. The colony was maintained in an insectarium under laboratory standard condition: temperature 25 ± 1°C; relative humidity 65 ± 5% and light–dark cycle of 16–8 hours. Mosquitoes were nourished with a 10% sucrose solution and fresh apple slices and, for reproduction, they were supplied with blood from cattle and poultry as described below for mosquito infection.

### *Dirofilaria immitis* microfilariae

The positive and negative canine blood samples for microfilariae (mf) were selected by one of the authors (AFR) during routine diagnostic duties at the Laboratory of Parasitology, Padua University. The infected dog was a 14 year old mixed breed, living outdoors in a village in north-eastern Italy, with no history of travel outside its native village. The presence of *D. immitis* in blood samples was confirmed by filtration test [23], serology test (SNAP Heartworm RT Test©, IDEXX, Laboratories, Inc., USA) and a duplex real-time PCR, as described elsewhere [18]. Sequencing of the positive blood sample confirmed the presence of *D. immitis* but not of *D. repens,* also reported in the area [17,18]. The blood was also screened by PCRs for other pathogens transmitted by arthropods known to be endemic in north-eastern Italy [24], i.e. *Leishmania infantum* [25], *Rickettsia* spp. [26], *Anaplasma phagocytophilum* [27], *candidatus Neoehrlichia mikurensis* [28], *Borrelia burgdorferi* sl [29] and *Babesia*/*Theileria* species [30].

Microfilariae were counted by examining thick blood smears by microscopy: ten drops of 20 μl were placed onto glass slides with a cover glass and examined under a light microscope (40×). The average number of mf was estimated by assessing the mean values observed in the ten counts [31]. Approximately 3000 mf per ml of blood were estimated.

### *Aedes koreicus* infection

Two experimental groups of mosquitoes were fed using blood with mf (n = 54; test group, T) or without mf (n = 29; control group, C), according to their availability. The mosquitoes were maintained in two cages measuring 50 cm per side for (T) and 30 cm per side for (C). The Density-Resting Surface (DRS) was calculated by dividing the vertical resting surface area of the cages (cm^2^) by the number of mosquitoes inside [32], being the final DRS values 185.2 in (T) and 124.1 in (C), corresponding to a low density. The mosquito population density into the cages was kept low to avoid mortality caused by crowding.

All mosquitoes were fed using an artificial feeding system (Hemotek® feeding system; Discovery Workshops, Lancashire, United Kingdom) [33] loaded with 5 ml of infected or negative blood with Na Citrate 9NC 3.8% anticoagulant solution in (T) and (C) group, respectively. The blood was provided for half an hour after which time unfed mosquitoes were removed from the cages. All bloodfed mosquitoes were provided with a 10% sucrose solution and fresh apple slices *ad libitum*.

Within 24 hours post- infection, three mosquito specimens were killed and dissected from (T) group to score the number of mf ingested. Mosquitoes were killed and examined at scheduled days (see Table 1). The mosquitoes were killed by putting them in dry ice and dissected in phosphate buffer saline (PBS) solution for the detection of *D. immitis* larvae under the stereomicroscope. All the mosquitoes naturally dead during the study were also examined. The head, the thorax and the abdomen of mosquitoes were examined on separate slides.

**Table 1:** **Specimens of** ***Aedes koreicus*** **examined for microfilariae (mf) and larval stages of** ***Dirofilaria immitis*** **at scheduled days post infection**

	**Total mosquitoes**							
	**Examined**	**Killed**				**Naturally dead**		
**Days post infection**		**total larval stages counted/mosquito infected; mean (range)**				**total larval stages counted/mosquito infected; mean (range)**		
		**mf**	**L1**	**L2**	**L3**	**mf**	**L1**	**L3**
1	3	43/3;14.3 (1–36)	0	0	0			
2	3					45/3;15.0 (10–22)	0	0
3	3					33/3;11.0 (4–17)	26/2;8.7 (0–22)	0
4	1					29/1	0	0
6	5					2/2;0.4 (0–1)	66/3;13.2 (0–26)	0
7	2					0	0	0
8	1					0	0	15/1
9	3					0	1/1	17/2;5.6 (0–10)
13	2*	0	0	8/1	7/1	0	0	3/1
16	1	0	0	0	15/1			
20	3					0	0	2/2;0.7 (0–1)
21	2					0	0	3/1;1.5 (0–3)
22	5	0	0	0	15/5;3.0 (0–4)			
24	1					0	2/1	1/1
27	3					0	0	0
28	4**	0	0	0	5/1	0	0	7/2;2.3 (0–4)
**Total**	42***	**43/3;14.3 (1–36)**	**0**	**8/1**	**42/8;5.2 (0–15)**	**111/9;8.5 (0–22)**	**93/7;8.4 (0–22)**	**48/10;3.4 (0–17)**

*one killed and one naturally dead, **one killed and three naturally dead, *** three specimens for histology not included (one killed and two dead at 6 dpi).

The larval developmental stages retrieved were identified, counted and, when possible, measured [34]. In addition five specimens, three from T group (two dead and one killed) and two from C group (one dead and one killed) were used for histological observations as described elsewhere [35,36].

After the microscopic observations, the head, thorax, and abdomen of each sample was recovered in PBS and a molecular analysis was performed to screen for the presence of *D. immitis* using a duplex real-time PCR [18].

### Statistical analysis

The rate of mortality was calculated each day, within 9 dpi and at the end of the study (28 dpi) as the percentage of dead mosquitoes over the total engorged in each cage (n = 42, excluded the three mosquitoes sacrificed for the mf intake calculation). The significance of the difference in mortality rates in the two cages was tested using the Fischer’s Exact test.

The Infection Rate (IR) and the Vector Efficiency Index (VEI) were calculated for (T) group [37]. The IR is defined as the number of blood fed mosquitoes showing infective third stage larvae (L3) in their body multiplied by 100 and divided by the number of surviving mosquitoes at the end of the incubation period. The period of extrinsic incubation is the number of days required for the development of the first L3 and was set arbitrarily at 12 dpi to be comparable with other studies [37]. The VEI is defined as the average number of L3 developed in the mosquitoes from the dpi of the first recovered L3 to the end of the study, multiplied by 100 and divided by the average number of ingested mf. The VEI was here determined in the period 8–28 dpi. The concordance between the finding of mf and larval stages by microscopy and PCR was calculated using the k coefficient (Win Epi 2.0 software, available online at: http://www.winepi.net/uk/index.htm).

## Results

After the blood meal, a similar percentage of mosquitoes were engorged in the two groups (i.e. 83.3% and 82.7% in T and C, respectively). The mean number of mf ingested per female in group T was 14.3 (range from 1 to 36).

**Figure 1:**
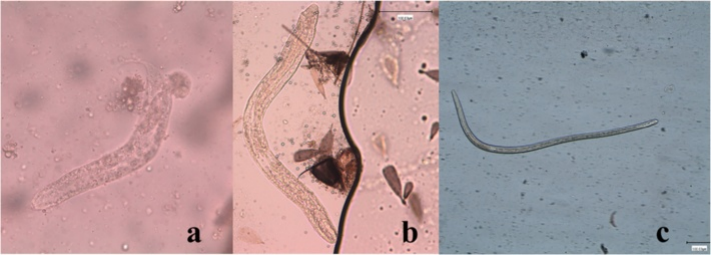
**Larval stages of** ***Dirofilaria immiti*** **s within** ***Aedes koreicus.*** **a)** L1 (40X); **b)** L2 (10X); **c)** L3 (5X).

The number of mf and larvae found during the study in killed and naturally dead mosquitoes is reported in Table 1. The larvae were identified as first stage larvae (L1), typically sausage-like in shape, second (L2) and third (L3) stage larvae (Figure 1), being their morphological features consistent with those reported in the literature [34] (Table 2). Movies of the living larval stages are accessible in Additional files 1, 2 and 3.

**Table 2:** **Parts of mosquitoes where microfilariae (mf) and larval stages of** ***Dirofilaria immitis*** **were found**

	**Total number larvae/mosquitoes (mean length μ)**			
**Body district**	**mf**	**L1**	**L2**	**L3**
abdomen	148/12 (301.5)	95/7 (190.0)	8/1 (309.6)	64/16 (986.5)
thorax	0/0	0/0	0/0	11/7 (889.6)
head	0/0	0/0	0/0	15/8 (932.1)

**Figure 2:**
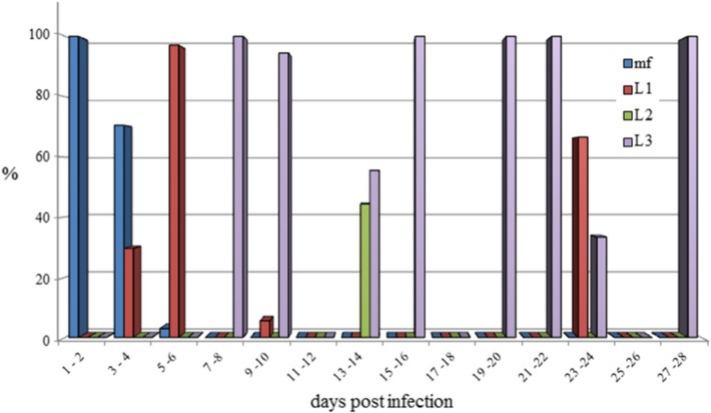
**Percentage of different larval stages of** ***Dirofilaria immitis*** **within** ***Aedes koreicus*** **mosquitoes.**

**Figure 3:**
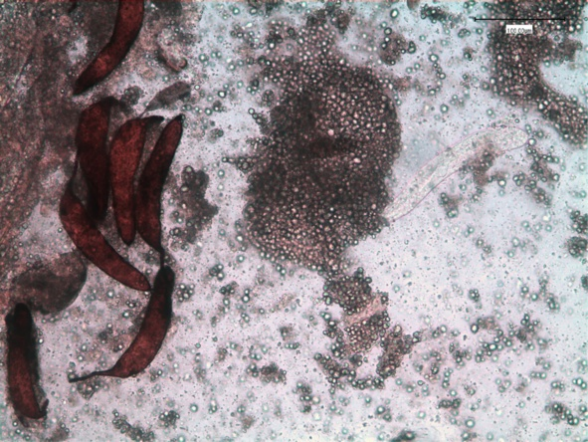
**L1 of** ***Dirofilaria immitis*** **alive (clear) and melanized (brown) within the abdomen of** ***Aedes koreicus*** **(20X).**

**Figure 4:**
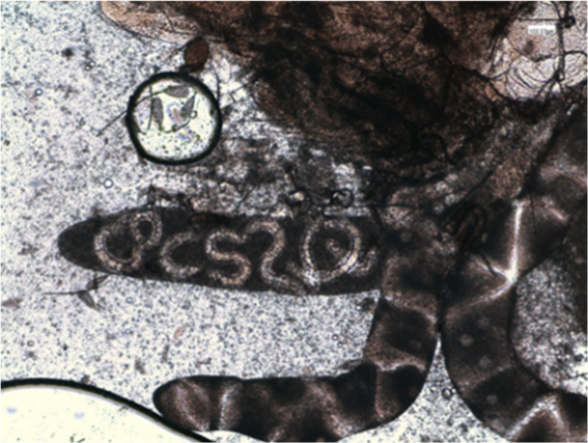
**L2 of** ***Dirofilaria immitis*** **within the Malpighian tubules of** ***Aedes koreicus*** **(5X).**

**Figure 5:**
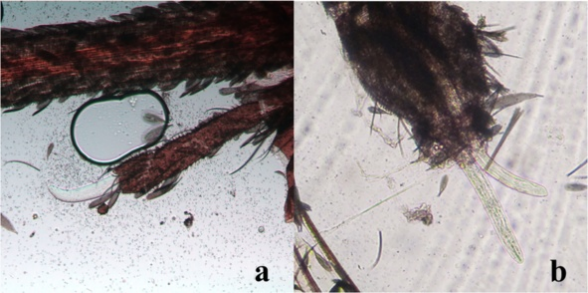
**L3 of** ***Dirofilaria immitis*** **within the head of** ***Aedes koreicus*** **(10X).** L3 emerging from the palp **(a)** and the proboscis **(b)**.

**Figure 6:**
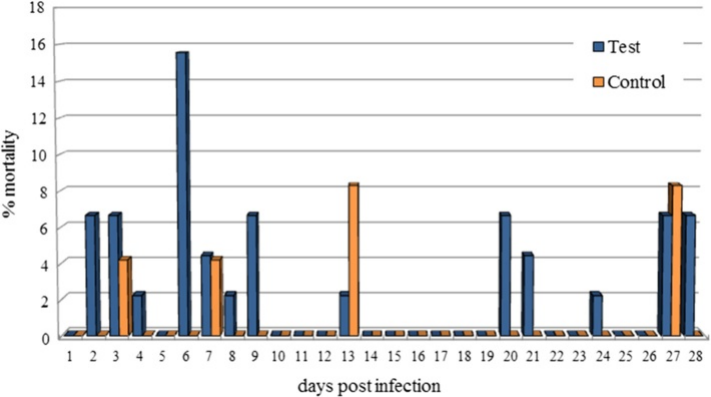
**Rate of mortality of** ***Aedes koreicus*** **infected with** ***Dirofilaria immits*** **during the study.**

Microfilariae were found in all the naturally dead mosquitoes until 6 dpi (Figure 2). L1, L2 and L3 were found in abdomen, whereas in the thorax and head only L3 were observed (Table 2). L1 were generally observed from 3 to 9 dpi with the exception of two L1 in a single mosquito at 24 dpi (Table 1). The number and the proportion of L1 were particularly high at 6 dpi (mean = 22.3; 97.1%), then rapidly declined due to development and mortality. Indeed L1 were found melanized and dead at 6 dpi (Figure 3). L2 (n = 8) were found only once in the Malpighian tubules of a mosquito at 13 dpi (Table 2, Figure 4). The first L3 were observed in the Malpighian tubules at 8 dpi (n = 15 in one mosquito) (Additional file 4) and, later on, from 16 through 28 dpi, they were also found in the thorax, salivary glands, palp and proboscis (Figure 5). In particular, L3 were observed emerging from the proboscis of three mosquitoes (Additional file 5). At the end of the study, 18 mosquitoes developed L3, giving an estimated IR of 68.2% and a VEI of 25.2%. The overall rate of mortality in mosquitoes was 78.6% (n = 33) and 25% (n = 6) in group T and C, respectively (p < 0.01), being higher during the first 9 dpi (i.e., 47.6% in group T vs 8.3% in group C; p < 0.01) (Figure 6).

Detection of *D. immitis* by molecular analysis showed a high but not complete agreement (k = 0.8-0.9) with microscopic identification, with PCR being slightly more sensitive than microscopy (Table 3). *D. immitis* was found in the head at 13 dpi by PCR, three days before it was observed by microscopy.

**Table 3:** **Concordance (k coefficient) between microscopy and PCR**

	**Positivity for** ***Dirofialria immitis***		
	**Microscopy neg/pos**	**PCR neg/pos**	**K (95% CI)**
Abdomen	11/31	8/34	0.8 (0.50-1.09)
Thorax	35/7	34/8	0.9 (0.62-1.22)
Head	34/8	31/11	0.8 (0.50-1.09)

All the mosquitoes tested by histology showed no larvae or evidence of alterations in their body.

## Discussion

Despite the fact that the experiment was not repeated due to the limited availability of mosquitoes, the finding of L3 emerging from the proboscis clearly indicated that *D. immitis* infective larvae developed in this mosquito species. Further experimental evidence of L3 transmission to a host is necessary for a final confirmation of the role *Ae. koreicus* plays as intermediate host for *D. immitis*.

The larval development to the infective third stage in other vectors of *D. immitis* (e.g. *Culex*, *Aedes*, *Ochlerotatus* and *Anopheles*) depends on several factors [34,38]. The ability of an invertebrate host to survive mf invasion and consequent development is pivotal for assessing its vector competence*.* Indeed, soon after ingestion, mf pass through the mosquito pharynx reaching the midgut. In about 24 hours they migrate to the Malpighian tubules where they develop into L1, L2 and finally L3 stages, eventually emerging from the tubules and migrating through the thorax to the salivary glands and the proboscis [38-41]. Accordingly, the critical period for the survival of *Ae. koreicus* (i.e. the higher rate of mortality) was during the first 4 dpi and around 8–9 dpi, corresponding to the invasion of the Malpighian cells by mf and to the emergence of L3 from the tubules, respectively.

Furthermore, the rate of survival of mosquitoes infected with *D. immitis* may be affected by the number of mf ingested, with high microfilaraemia causing fatal injuries leading to mortality [39,42,43]. The high overall rate of mortality of *Ae. koreicus* herein recorded (78.6%) is likely affected by the length of the study. Indeed, the rate of mortality within the first 9 dpi (47.6%) was similar to that of other studies carried out for *Ae. aegypti* (48.1%; 9 dpi) and for different strains of *Ae. albopictus* (32.9-54%; 15 dpi), where mosquitoes were experimentally administered with a comparable number of mf (i.e., 3000–3300 mf/ml) [43,44]. Accordingly, if *Ae. koreicus* is able to support the development of infective L3 at higher levels of microfilaraemia remains to be demonstrated, since dogs harboring high (35,000 mf/ml) to very high (>70,000 mf/ml) numbers of mf may be a frequent occurrence in geographical areas endemic for canine heartworm disease [17,19,45].

In our study the observation of melanized L1 only once within 6 dpi suggests that the majority of them were not destroyed. Previous studies on *Ae. albopictus* infected with *D. immitis* reported the arrest of the development of L1 and L2 stages, i.e. melanization and degeneration of larvae in the Malpighian tubules [44,46,47], or the arrest of the development at the mf stage in *Ae. aegypti*, [38,48,49]. In *Ae. koreicus* Feng [21] observed degenerated larvae at different stages but especially as mf.

Importantly, artificial blood-feeding, incorporating anticoagulants, might increase the rate of mf migration, because blood clotting in the midgut has been found to reduce mf migration to the Malpighian tubules [38,48,50].

The minimum extrinsic incubation period herein observed (8 dpi) is consistent with the previous finding in *Ae. koreicus* [21]. The timing of L3 recovery from the head of *Ae. koreicus* by Feng [21] (i.e. between 10 and 15 dpi) nicely overlaps with our molecular identification, as well as the time of our first observations of L3 by microscopy (i.e., 16 dpi) is similar to that of *D. immitis* in *Ae. albopictus* [46,47].

The likely role of *Ae. koreicus* as a vector of *D. immitis* is also corroborated by our IR and VEI values (68.2% and 25.2%, respectively). Indeed, a mosquito species is suggested to be “susceptible” to infection when IR and VEI values are above 10% and 8-9%, respectively [38]. By those criteria *Ae. aegypti* fed with blood containing 3,000 mf/ml and showing IR = 55.3% and VEI = 6.3% was considered as “refractory” [43] whereas *Ae. albopictus* fed with blood containing 3,490 mf/ml and showing IR = 28% and VEI = 45.8% was considered susceptible [31].

The risk of canine and human infection by *D. immitis* in a given area is primarily dependent on the presence of competent mosquito vectors and microfilaremic dogs, although other factors are involved, such as the density of vectors, their host-seeking activity and feeding preference [19,51]. Early studies carried out in north-eastern Italy [13] have shown that *Ae. koreicus* is well- adapted to urban settlements. The species is found in sympatry with *Ae. albopictus*, but also in other empty niches. Indeed, the highest density of *Ae. koreicus* is found in hilly and mountainous habitats, poorly or not yet colonized by *Ae. albopictus* [13]. Laboratory and field studies suggest a preference of *Ae. koreicus* for humans, however, the species was able to complete its life cycle when fed with canine blood. Dog blood has also been found in a specimen captured from the field [52]. All these factors, coupled with the likely vector competence of *Ae. koreicus* for *D. immitis,* may increase the risk of exposure to *D. immitis* for dogs and humans in areas where dirofilariosis is present by extending it to areas previously considered at negligible risk in Italy and Europe [19,53].

Further studies on the vector competence of *Ae. koreicus* for pathogens such as dengue and chikungunya viruses, *D. repens* and other filarioids are warranted in order to better understand the role played by this recently imported mosquito species in the epidemiology of the diseases they may transmit to animals and humans.

## Conclusion

In conclusion, *Aedes koreicus*, a new invasive species for Europe, is most likely a competent vector of *D. immitis,* being of potential relevance in the natural cycle of the parasite. This poses a new threat for animal and human health in endemic areas for dirofilariosis and enhances the risk of spreading the infection in previously non-endemic areas. It is important that veterinarians and physicians are aware of the presence of a new competent vector of *D. immitis* in areas where the risk of dirofilariosis for pets and humans was previously considered negligible. These results stress the importance of active surveillance and control strategies to minimize the risk of introduction of invasive alien species.

## Additional files

Additional file 1:
***Dirofilaria immitis*** **larvae L1 in the** ***Aedes koreicus*** **abdomen.**
Additional file 2:
***Dirofilaria immitis*** **larvae L2 in the** ***Aedes koreicus*** **abdomen.**
Additional file 3:
***Dirofilaria immitis*** **larvae L3 in the** ***Aedes koreicus*** **abdomen.**
Additional file 4:
***Dirofilaria immitis*** **larvae L3 in the** ***Aedes koreicus*** **Malpighian tubules.**
Additional file 5:
***Dirofilaria immitis*** **larvae L3 emerging from the** ***Aedes koreicus*** **proboscis.**
